# Effect of glycemic control and type of diabetes treatment on unsuccessful TB treatment outcomes among people with TB-Diabetes: A systematic review

**DOI:** 10.1371/journal.pone.0186697

**Published:** 2017-10-23

**Authors:** Hemant Deepak Shewade, Kathiresan Jeyashree, Preetam Mahajan, Amar N. Shah, Richard Kirubakaran, Raghuram Rao, Ajay M. V. Kumar

**Affiliations:** 1 International Union Against Tuberculosis and Lung Disease (The Union), South-East Asia Office, New Delhi, India; 2 International Union Against Tuberculosis and Lung Disease (The Union), Paris, France; 3 Velammal Medical College Hospital & Research Institute, Madurai, India; 4 All India Institute of Medical Sciences (AIIMS), Bhubaneswar, India; 5 U.S. Agency for International Development (USAID), American Embassy, New Delhi, India; 6 Cochrane South Asia, Christian Medical College, Vellore, India; 7 Central TB Division, Revised National Tuberculosis Control Programme, Ministry of Health and Family Welfare, Government of India, New Delhi, India; Saint Louis University, UNITED STATES

## Abstract

**Background:**

**S**tringent glycemic control by using insulin as a replacement or in addition to oral hypoglycemic agents (OHAs) has been recommended for people with tuberculosis and diabetes mellitus (TB-DM). This systematic review (PROSPERO 2016:CRD42016039101) analyses whether this improves TB treatment outcomes.

**Objectives:**

Among people with drug-susceptible TB and DM on anti-TB treatment, to determine the effect of i) glycemic control (stringent or less stringent) compared to poor glycemic control and ii) insulin (only or with OHAs) compared to ‘OHAs only’ on unsuccessful TB treatment outcome(s). We looked for unfavourable TB treatment outcomes at the end of intensive phase and/or end of TB treatment (minimum six months and maximum 12 months follow up). Secondary outcomes were development of MDR-TB during the course of treatment, recurrence after 6 months and/or after 1 year post successful treatment completion and development of adverse events related to glucose lowering treatment (including hypoglycemic episodes).

**Methods:**

All interventional studies (with comparison arm) and cohort studies on people with TB-DM on anti-TB treatment reporting glycemic control, DM treatment details and TB treatment outcomes were eligible. We searched electronic databases (EMBASE, PubMed, Google Scholar) and grey literature between 1996 and April 2017. Screening, data extraction and risk of bias assessment were done independently by two investigators and recourse to a third investigator, for resolution of differences.

**Results:**

After removal of duplicates from 2326 identified articles, 2054 underwent title and abstract screening. Following full text screening of 56 articles, nine cohort studies were included. Considering high methodological and clinical heterogeneity, we decided to report the results qualitatively and not perform a meta-analysis. Eight studies dealt with glycemic control, of which only two were free of the risk of bias (with confounder-adjusted measures of effect). An Indian study reported 30% fewer unsuccessful treatment outcomes (aOR (0.95 CI): 0.72 (0.64−0.81)) and 2.8 times higher odds of ‘no recurrence’ (aOR (0.95 CI): 2.83 (2.60−2.92)) among patients with optimal glycemic control at baseline. A Peruvian study reported faster culture conversion among those with glycemic control (aHR (0.95 CI): 2.2 (1.1,4)). Two poor quality studies reported the effect of insulin on TB treatment outcomes.

**Conclusion:**

We identified few studies that were free of the risk of bias. There were limited data and inconsistent findings among available studies. We recommend robustly designed and analyzed studies including randomized controlled trials on the effect of glucose lowering treatment options on TB treatment outcomes.

## Introduction

Tuberculosis (TB) remains a major public health problem in low and middle income countries. [1] Meanwhile, the burden of diabetes mellitus (DM) is increasing to epidemic proportions in the same countries.[2,3] Worldwide, there are an estimated 10.4 million new people with active TB annually and of them, one million have both TB and DM (TB-DM). This double burden deserves attention. [2–4]

DM increases the risk of incidence of TB by 2–3 folds and bi-directionality of association between TB and DM has been studied. [5–7] Although there is no singular mechanism that has been identified as the cause of this bi-directional association, immune compromise is widely accepted as the reason for increased risk of TB among DM; and inflammation (mediated by IL6 and TNFα) whilst modulating a response to TB infection could cause an increase in insulin resistance. [8] Risk of TB is higher in DM patients with ‘poor glycaemic control’ as compared to those with ‘optimal glycaemic control’. [9–11] Among people with TB, DM increases the risk of unfavourable treatment outcomes (delayed culture conversion, death, treatment failure, recurrence). [12,13] Hyperglycemia among people with TB is associated with more severe clinical manifestations during TB treatment like higher bacterial load in sputum, increased leucocyte count, increased acute phase response, more fever and atypical localization and cavity formation. [14–19] Glycemic control results in improvement in phagocytic activity [20], and avoidance of above-listed clinical complications. There is also evidence that enhanced management of DM reduces the risk of developing TB and improving TB treatment outcomes. [21]

To address the looming TB-DM epidemic, World Health Organization (WHO) and International Union Against Tuberculosis and Lung Disease (The Union) developed a collaborative framework in 2010 for care and control of TB and DM. [22]

### DM management among TB

Goals for glycemic control among people with DM are defined using various biochemical tests such as fasting/pre-prandial capillary blood glucose (FBG) or 2 hour post-prandial capillary blood glucose (PPBG) or glycosylated hemoglobin (HbA1c). Corresponding less stringent glycemic goals using higher cut offs of FBG, PPBG and HbA1c are available for those with extensive co-morbid conditions, less life expectancy and extensive micro/macro vascular complications. The same may be applied to TB people with DM. [23]

Rifampicin and Isoniazid have been documented to interact with OHAs and hamper glycemic control. [24] Insulin is more efficacious than OHAs in achieving glycemic control, though, the chances of hypoglycemic episodes increase with insulin use.[25,26] Using insulin treatment to replace or add to OHAs when TB is diagnosed among people with DM has been recommended by some for better glycemic control especially in severe tuberculosis. [24]

### Why is it important to do this review?

There has been a call for integrating communicable and non-communicable disease care. [27] One of the four high priority research agendas recommended for reducing the joint burden of TB-DM has been to determine the impact of glucose lowering treatment on TB treatment outcomes with a detailed assessment on death. [28]

Though stringent glycemic control and using insulin as a replacement or in addition to OHAs have been recommended when DM is diagnosed in people with TB,[22,24,29,30] there is a need to systematically review whether this actually leads to improved TB treatment outcomes. Jorgensen et al found studies with mixed reports regarding effect of glycemic control on TB treatment outcomes. Some of the results quoted were not statistically significant, quality of studies included was not assessed and pooled estimates were not available. [31]

**Specific objectives**: Among people with drug susceptible TB and DM on anti-TB treatment (ATT), ***primary objective*** was to determine the effect of the following on unsuccessful TB treatment outcome(s) i) glycemic control (stringent or less stringent) when compared to poor glycemic control ii) insulin, alone or in combination with OHAs when compared to OHAs only. ***Secondary objectives*** were to determine the i) effect of glycemic control on emergence of multi-drug resistant TB (MDR-TB) at the end of TB treatment and recurrence of TB after successful treatment completion (after 6 months, after 1 year and beyond), ii) association of type of DM treatment and adverse outcomes (including hypoglycemic episodes) related to glucose lowering treatment.

The findings from this systematic review may provide evidence and guide the existing programmes, especially in low and middle income countries (with a high burden of TB-DM), regarding development of guidelines for management of DM during ATT and also guide future research priorities in TB-DM.

## Material and methods

### Protocol

The protocol was registered with PROSPERO (PROSPERO 2016:CRD42016039101) and is available online. There were no protocol deviations. The search is up to date as on 25 April 2017. [32]

### Inclusion criteria for studies

#### Types of studies

We intended to include all interventional studies (with a control arm) on the topic (randomized or non-randomized; individual or cluster randomized) and all cohort studies (retrospective, prospective and ambispective). All studies between 1996 and 25 April 2017, published in any country and any language were included. We excluded single arm intervention studies, before-after design without control, cross-sectional studies, case control studies, case series, case reviews, subject reviews and ecological studies.

#### Types of participants

Participants were people of all ages and sexes with TB on ATT (not known to be drug-resistant at baseline) and diagnosed with diabetes (type I or type II or any other type) before or during TB diagnosis or during ATT. We included people on daily or intermittent anti-TB regimens (at least Isoniazid, Rifampicin, Pyrazinamide, Ethambutol); with or without HIV; managed either in programmatic or clinical (public or private facility) settings; managed in inpatient or outpatient settings; with pulmonary or extra pulmonary TB; with or without cavities at baseline chest radiograph (in case of pulmonary TB); with microbiologically or clinically diagnosed TB; and with new or retreatment TB.

#### Types of interventions / exposure

For primary and secondary objective one, ‘stringent glycemic control’ was pre-defined based on one time measurement of capillary FBG of <130 mg/dl (7.2 mmol/l) or capillary PPBG <180 mg/dl (10mmol/l) or HbA1c of <7%. Corresponding less stringent glycemic goals were capillary FBG of <178 mg/dl (9.9 mmol/l) or capillary PPBG <206 mg/dl (11.4 mmol/l) or HbA1c of <8%. [25,33] Glycemic control could be defined at various TB treatment phases: at baseline or any time during treatment (before / after intensive phase). People with ‘poor glycemic control’ were the comparison group.

For primary and secondary objective two, people on various glucose lowering treatment options (allopathic) were included. Individual level pharmacological interventions or exposures were included. The various glucose lowering treatment options could be ‘insulin only’, ‘insulin with OHAs’ or ‘OHAs only’. People receiving ‘OHAs only’ were the comparison group.

#### Types of outcome measures

Primary outcomes were TB treatment outcomes, classified as favourable and unfavourable at the end of intensive phase and / or at end of TB treatment (minimum six months and maximum 12 months follow up). (Table 1) [34] Secondary outcomes were development of MDR-TB during the course of treatment, recurrence during follow up after 6 months and/or after 1 year post successful treatment completion and development of adverse event related to glucose lowering treatment (including hypoglycemic episodes).

**Table 1:** Operational definitions of TB treatment outcomes: end of intensive phase and end of treatment [34].

**At the end of treatment**	
Cured	A pulmonary TB patient with bacteriologically-confirmed TB at the beginning of treatment who was smear- or culture-negative in last month of treatment and on at least one previous occasion
Treatment completed	A TB patient who completed treatment without evidence of failure, but with no record to show that sputum smear or culture results in the last month of treatment and on at least one previous occasion were negative, either because tests were not done or because results are unavailable.
Lost to follow-up	A TB patient who did not start treatment or whose treatment was interrupted for two consecutive months or more.
Treatment failed	A TB patient whose sputum smear or culture is positive at month five or later during treatment.
Died	A TB patient who dies for any reason before starting or during the course of treatment
Not evaluated	A TB patient for whom no treatment outcome is assigned. This includes cases “transferred out” to another treatment unit as well as cases for whom the treatment outcome is unknown to the reporting unit.
Unfavourable outcome	Died, treatment failed, lost to follow-up, not evaluated
Favourable outcome	Treatment completed, cured
**At the end of intensive phase** (2 months for new patient, 3 months for a previously treated patient)	
Unfavourable outcome	Died, Lost to follow-up, extension of intensive phase and non-conversion at three/four months
Favourable	Microbiological conversion

### Search methods for identification of studies

#### Electronic searches

We searched separately for interventional and cohort studies in PubMed, EMBASE, Google Scholar and the Cochrane Database of Systematic Reviews (search strategy and search results in S1 Appendix).

#### Searching other resources

We looked for cross references in the included studies, and reports; online and offline. We communicated with relevant experts in the fields for any article/research study (ongoing or recently completed) on this topic. A grey literature search included ISI Web of Science with conference proceedings, ClinicalTrials.gov, national and international trials registers, the World Health Organization (WHO) International Clinical Trials Registry Platform (ICTRP) search portal (apps.who.int/trialsearch/), metaRegister of Controlled Trials (mRCT) (controlledtrials.com/mrct/) and trial results registers, and guidelines and their reference lists. We contacted Jorgensen et al for the list observational studies they had listed for their review. [31]

### Data collection and analysis

#### Selection of studies

We removed duplicates and imported the bibliography to Rayyan for initial screening of title and abstracts. Rayyan is an open access web application (https://rayyan.qcri.org/). Once the bibliography was uploaded, it facilitated independent screening of bibliographic records (with blinding) followed by a providing a summary of screening (number of records included or excluded with consensus and number with no consensus). [35] During full text screen, the study was included for data extraction only if it fulfilled all the criteria (study type, participant, intervention/exposure with comparator, and outcome). If the study did not meet any one of the above criteria, it was excluded. Screening was done by two investigators (KJ and PM) independently. The investigators resolved any disagreements by mutual consent; with recourse to a third investigator (ANS for title/abstract screen; HDS for full text screen), if required.

#### Data extraction and management

Two investigators (KJ and PM) independently extracted study data from full text of the included studies into a data extraction form. (S2 Appendix) Any disagreement was resolved by discussion with a third investigator (HDS).

If primary data were unavailable in the supplementary file and/or no effect measures (adjusted/unadjusted) were reported, we requested the same form the authors. We sent three email reminders, fortnightly and awaited response for a maximum period of 1 month after writing the first mail to them.

#### Assessment of risk of bias in included studies

For interventional study, we planned to rate each included trial as being at high, low or unclear risk of bias under the following domains: sequence generation, allocation concealment, blinding of participants, personnel and outcome assessors, incomplete outcome data, selective outcome reporting and other sources of bias like carry-over, recruitment bias and contamination. [36] For cohort studies, we modified New Castle-Ottawa quality assessment scale and summarized it in a risk of bias table. [37]

#### Measures of effect

We planned to report the effect separately for interventional and cohort studies using adjusted or unadjusted Odds Ratio (OR/aOR) or Relative Risk (RR/aRR) or Hazard Ratio (HR/aHR) along with 0.95 confidence interval (CI). If the desired adjusted comparison was not available, the authors calculated the unadjusted effect based on the data available from narrative texts/tables. If primary data were available, we planned to perform analysis after appropriate adjustment followed by a pooled analysis, if possible.

#### Assessment of reporting biases

Considering possible reporting bias we contacted the study authors to get more information about the reported outcome. We also contacted the authors of registered trials/studies (but not yet published) in registries. Subject to inclusion of sufficient studies, we planned to plot the funnel plot to detect publication bias.

We followed the Preferred Reporting Items for Systematic reviews and Meta-Analyses (PRISMA statement) and Meta-analysis Of Observational Studies in Epidemiology (MOOSE) checklist to report this review. [38,39]

## Results

**Fig 1:**
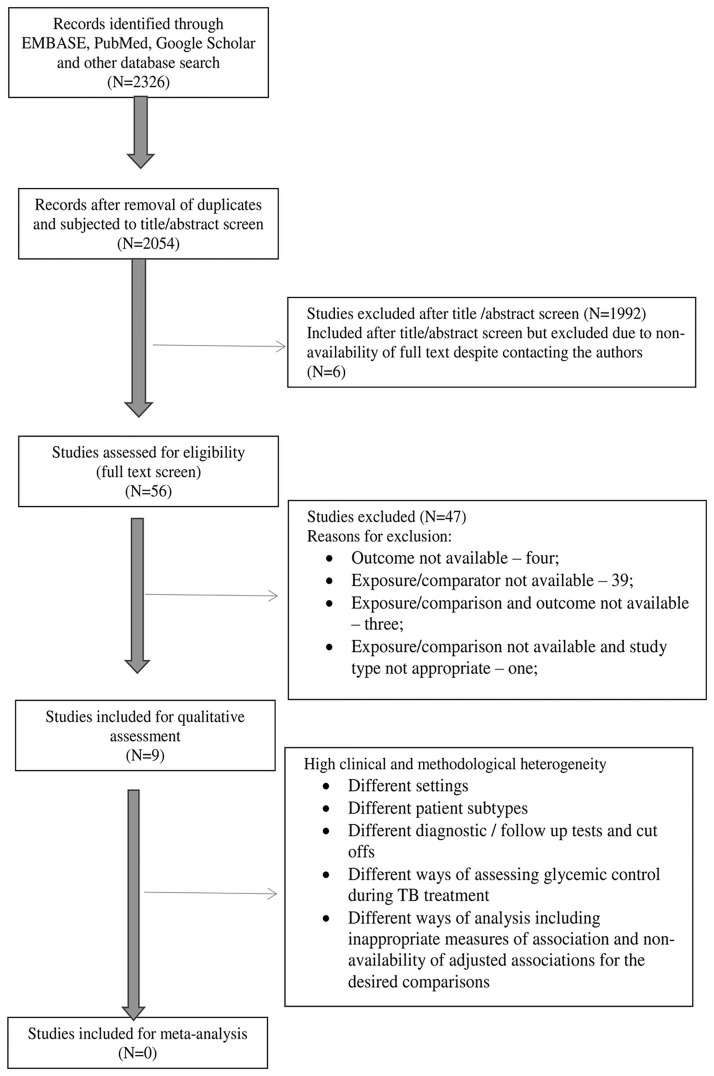
PRISMA flow diagram through different phases of the systematic review [39].

We found 2326 articles after database search. After removal of duplicates, 2054 underwent a title and abstract screen and 56 were assessed for eligibility (full text screen). Nine studies were included for qualitative assessment (data extraction). Considering high methodological and clinical heterogeneity, we decided to report the results qualitatively. (Fig 1) Reasons for exclusion of studies during full text screen have been reported in Table 2. Characteristics of included studies are presented in S1 Annex.

**Table 2:** Characteristics of the studies excluded from the systematic review*.

S.No	Study identifier	T	P	E/C	O
1.	Hongguang C_2015_Epidemiol Infect	Y	Y	N	Y
2.	Wang CS_2009_Epidemiol Infect	Y	Y	N	Y
3.	Banurekha VV_2007_IJMR	Y	Y	N	Y
4.	Chang JT_2011_J Formos Med Assoc	Y	Y	N	Y
5.	Kota SK_2011_Diabetes and Metabolic Syndrome Clinical Research and Reviews	Y	Y	N	Y
6.	Duangrithi D_2013_International J of Clinical Practice	Y	Y	N	Y
7.	Fielder JF_2002_Int J of Tub Lung Dis	Y	Y	N	Y
8.	Jabbar A_2006_Eastern mediterranean health Journal	Y	Y	N	Y
9.	Perez-Navarro LM_2015_J of Diabetes and its complications	Y	Y	N	Y
10.	Iseri AU_2010_Tüberküloz veToraksDergisi	Y	Y	N	Y
11.	B.E. Abdelbary_2016_Tuberculosis	Y	Y	N	Y
12.	Bachti Alisjahbana_2007_Clinical Infectious Diseases	Y	Y	N	Y
13.	Rani Balasubramanian_2007_Indian J of TB	Y	Y	N	Y
14.	Boillat_Blanco N_2016_The J of Infectious Diseases	Y	Y	N	Y
15.	Dobler CC_2012_BMJ Open	Y	Y	N	N
16.	Castellanos-Joya M_2014_Plos One	Y	Y	N	Y
17.	Chaudhry LA_2012_International J of Mycobacteriology	Y	Y	N	Y
18.	Faurholt-Jepsen D_2012_BMC Infectious Disease	Y	Y	N	Y
19.	Faurholt-Jepsen D_2013_TMIH	Y	Y	N	Y
20.	Suwampimolkul G_2014_Plos One	Y	Y	N	Y
21.	Gnanasan S_2012_University of Nottighamphd thesis	Y	Y	N	N
22.	Johnson HD_2016_American Journal of Infectious Disease and Microbiology	Y	Y	N	Y
23.	Dooley KE_2009_Am J Trop Med Hyg	Y	Y	N	Y
24.	Wang JY_2015_Chest	Y	Y	N	Y
25.	Lee PH_2016_Plos Medicine	Y	Y	Y	N
26.	Lo HY_2016_Int J of Tub Lung Dis	Y	Y	N	Y
27.	Wang JY_2013_Pharmacoepidemiology and drug safety	Y	Y	N	N
28.	Workneh MH_2016_Infectious Disease of Poverty	Y	Y	N	Y
29.	Oceguera DM_2016_Lung Disease and treatment	Y	Y	N	Y
30.	Orofino RDL_2012_J Bras Pneumol	Y	Y	N	Y
31.	Pajankar S_2008_Oman Medical Journal	Y	Y	N	Y
32.	Sahakyan S_2015_not a peer reviewed publication	Y	Y	N	Y
33.	Jimenex-Corona ME_2013_Thorax	Y	Y	N	Y
34.	Shariff NM_2015_Int J of mycobacteriology	N	Y	N	Y
35.	Sulaiman SAS_2013_American J of medical sciences	Y	Y	N	Y
36.	Vellalacheruvu BN_2015_International J of Scientific and Research Publications	Y	Y	N	Y
37.	Wang JY_2009_Respirology	Y	Y	N	Y
38.	Yusupova S_2016_Public Health Panorama	Y	Y	N	Y
39.	Siddiqui AM_2009_Journal of Taibah University Medical Sciences	Y	Y	N	Y
40.	Kornfield H_2016_Chest	Y	Y	N	Y
41.	Gil_Santana L_2016_Plos One	Y	Y	N	Y
42.	Mukhtar F_2016_BMJ Open	Y	Y	N	Y
43.	Salindri AD_2016_Open Forum Infectious Diseases	Y	Y	N	Y
44.	Barss L_2016_Chest	Y	Y	N	Y
45.	Wu Z_2016_J of Diabetes and its complication	Y	Y	N	Y
46.	Lee EH_2017_Lung	Y	Y	N	Y
47.	Perez-Navarro LM_2017_Tuberculosis	Y	Y	N	Y

*T: Type of study = all interventional studies on the topic (randomized or non-randomized; individual or cluster randomized) with a control arm. Among observational studies, cohort studies (retrospective, prospective and / ambispective); P: participant criterion = people with TB and DM on anti-TB treatment; E/C: exposure/comparator = for research question on glycemic control, glycemic status was the exposure of interest. For research question on insulin, type of DM treatment was the exposure of interest. If any one of the two was present in the study we included it; O: outcome = the study was included if any one of the primary outcomes were present; Y—yes; N—no

Of the nine studies included in the review, all were cohort studies: eight dealt with glycemic control and its effect on TB treatment outcomes (Table 3) [40–47] and two dealt with type of DM treatment and TB treatment outcomes. (Table 4) [47,48] All studies included both sexes and adults, with children being included in one.[43] It was unclear whether children were included in the other two studies. [40,46] Four studies included patients with and without HIV, [40,42,44,47] three studies excluded patients with HIV [41,45,46] and in the remaining two information on HIV status was not available [43,48]. None of the authors responded with primary data or unreported outcomes. Hence, we did not perform any primary data analysis for adjusted effect measures. We derived unadjusted RRs from the data extracted from narrative texts/tables. As sufficient number of studies under each primary objective was less than 10, we did not plot the funnel plot to detect publication bias.

**Table 3:** Effect of glycemic control (stringent or less stringent) on unfavourable TB treatment outcomes, summarized as unadjusted relative risk (RR)* [40–47].

Study ID	Reference group	Exposed group	Unadjusted RR	95% CI
**All unfavourable end (TB) treatment outcome**				
Chiang CY_2015_Plos One	Poor glycemic control (HbA1c>9)	Less stringent glycemic control (HbA1c 7–9)	1.53	0.96, 2.46
		Stringent glycemic control (HbA1c<7)	1.89	1.12, 3.20
Mi F_2013_TMIH^	Poor glycemic control (FPG>10mmol/l)	Less stringent glycemic control (FPG7-10mmol/l)	0.91	0.18, 4.43
		Stringent glycemic control (FPG<7mmol/l)	1.03	0.21, 5.07
Magee MJ_2013_International J of Infectious diseases	Poor glycemic control (no specific criteria)	Glycemic control	1.08	0.54, 2.16
Nandakumar KV_2013_Plos One	Poor glycemic control (FBG>100 mg/dl and PPBS/RBG>140 mg/dl)	Glycemic control	0.52	0.25, 1.07
Tabarsi P_2014_Journal of Diabetes and Metabolic Disorder	Poor glycemic control (HbA1c≥6.5)	Glycemic control	1.13	0.2, 6.44
Yoon YS_2017_Thorax	Poor glycemic control (HbA1c≥7)	Glycemic control	0.55	0.22, 1.36
Mahishale_2017_Iran J MS	Poor glycemic control (HbA1c≥7)	Glycemic control	0.18	0.09, 0.36
**Culture non-conversion at 2 months**				
Park SW_2012_Eur J ClinMicrobiol Infect Dis	Poor glycemic control (HbA1c>7)	Glycemic control	0.62	0.14, 2.71
Yoon YS_2017_Thorax	Poor glycemic control (HbA1c≥7)	Glycemic control	0.23	0.05, 0.94
**Sputum smear non-conversion at 2 months**				
Mi F_2013_TMIH^	Poor glycemic control (FPG>10mmol/l)	Less stringent glycemic control (FPG7-10mmol/l)	1.50	0.57, 3.90
		Stringent glycemic control (FPG<7mmol/l)	0.65	0.19, 2.15
Nandakumar KV_2013_Plos One	Poor glycemic control (FBS>100 mg/dl and PPBS/RBS>140 mg/dl)	Glycemic control	0.99	0.65, 1.51
Mahishale_2017_Iran J MS	Poor glycemic control (HbA1c≥7)	Glycemic control	0.12	0.06, 0.23

*data extracted from the narrative text/tables;

^End IP and end treatment outcomes reported among those with glycemic status at 2 months and 6 months respectively

**Table 4:** Effect of glucose lowering treatment on unfavourable TB treatment outcomes, summarized as unadjusted relative risk (RR)* [47,48].

Study ID	Reference group	Exposed group	Unadjusted RR	95% CI
All unfavourable end (TB) treatment outcome				
Magee MJ_2013_International J of Infectious diseases	OHA only	Insulin	2.63	1.07, 6.46
		OHA+Insulin	0.81	0.23, 2.80
Viswanathan V_2014_Journal of Diabetes and its complications ^	-	-	-	-

*data extracted from the narrative text/tables;

^Unsuccessful TB treatment outcomes among those on OHA only, insulin only and both were 0/53, 2/18 and 0/3 respectively. Sufficient outcomes were not there to calculate RR.

### Glycemic control and TB treatment outcomes

Mahishale et al from India reported that compared to poor glycemic control (HbA1c ≥7%) at baseline, optimal glycemic control (HbA1c <7%) at baseline resulted in 88% reduction in sputum smear non-conversion at 2 months (unadjusted RR (0.95 CI):0.12 (0.06−0.23)); 30% reduction in unsuccessful treatment outcomes (aOR (0.95 CI):0.72 (0.64−0.81)) and 2.8 times higher odds of ‘no recurrence’ (aOR (0.95 CI):2.83 (2.60−2.92)). MDR-TB, confirmed by either culture or by Xpert MTB/RIF assay, was noted in 47 of 423 (11%) people with poor glycemic control as against none with optimal control group. [46] (Table 3, S1 Annex) Nandakumar et al, from India, found no association between glycemic control and end treatment outcomes. Glycemic control was considered as ‘known’ if FBG/RBG was available on three occasions, separated by one month and at least one of them was in continuous phase of ATT. If all three values were below the cut off (FBG<100mg/dl; RBG<140mg/dl), then it was considered as ‘optimal glycemic control’ during ATT. [44]

Chiang CY et al from Taiwan reported that stringent glycemic control (HbA1c <7%) at baseline was associated with (unadjusted RR (0.95 CI): 1.89 (1.12−3.20)) higher risk of unfavourable treatment outcome when compared to poor glycemic control (HbA1c ≥9%); while less stringent glycemic control (HbA1c 7–9%) was not (unadjusted RR (0.95 CI): 1.53 (0.96−2.46)). In the adjusted analysis, while DM was not associated with unfavourable TB treatment outcomes, DM related comorbidity was. [40](Table 3, S1 Annex)

Mi F et al from South China reported no significant association between stringent glycemic control (FPG<7 mmol/l) and less stringent glycemic control (FPG 7–10 mmol/l) with end IP and end treatment outcomes. Though FPG values were available at baseline, 2 months and 6 months, 2 months glycemic status was compared with end IP outcomes and 6 month glycemic status was compared with end treatment outcomes. [43] (S1 Annex)

Magee MJ et al from Lima, Peru found that among TB-DM patients (30% had drug resistance), culture conversion among those with glycemic control (assessed while on ATT) was faster than those without control. (aHR (0.95 CI) = 2.2 (1.1,4)). [42](S1 Annex)

Yoon YS et al from South Korea, used HbA1c <7% and ≥7% for glycemic control and poor glycemic control respectively in the adjusted analysis. However, people without DM were the reference. We extracted numbers for treatment outcomes among the subgroups of glycemic control in people with TB-DM. Culture non-conversion, but not end treatment outcomes, was associated with glycemic control when compared to poor control at baseline (unadjusted RR (0.95 CI):0.23 (0.05−0.94)). [41](Table 3, S1 Annex)

Data extracted from the study by Park SW et al from South Korea revealed no association between sputum conversion at 2 months and glycemic control (HbA1c<7) at baseline.[45] Though Tabarsi P et al did not exactly address our research question(s), we could extract the data that we required. HbA1c was measured at baseline and 3 months of ATT and was categorized as elevated if HbA1c was ≥6.5%. Those with ‘Elevated-normal’ and ‘elevated-elevated’ at baseline and 3 months respectively were included in our analysis to represent good control and poor control respectively. We found no significant difference in end treatment outcomes among these two groups.[47] (Table 3, S1 Annex)

### Type of DM treatment and TB treatment outcomes

Data extracted from Magee MJ et al from Lima, Peru revealed that when compared to those receiving ‘OHA only’, those receiving ‘Insulin only’ had significantly higher risk of unfavourable end treatment outcomes (unadjusted RR (0.95 CI):2.63 (1.07−6.47)); while those receiving ‘insulin and OHA’ did not have significantly different unfavourable end treatment outcomes.[42] (Table 4, S1 Annex) Viswanathan V et al did not have sufficient number of unfavourable outcomes to enable calculation of RR.[48] (Table 4, S1 Annex)

### Risk of bias assessment

Of the nine studies included, six had poor ‘comparability’, four of which did not report adjusted analysis. Four studies suffered inadequacies in follow up that was likely to induce bias. The study by Mahishale et al had no risk of bias. (Table 5)

**Table 5:** Summary of risk of bias in included studies assessed using New Castle Ottawa quality assessment scale for cohort studies [37].

Study ID	Selection (max 4 stars)	Comparability (max 3 stars)	Outcome (max 2 stars)	Overall comment
Chiang CY_2015_Plos One	****	-	**	Adjusted analysis done but for not of the comparison of our interest, glycemic control was not included in the adjusted analysis
Mi F_2013_TMIH	***	-	*	No description of derivation of TB DM glycemic control/uncontrolled cohort, cross sectional data used for analysis, no adjustment, incomplete follow up likely to introduce bias
Magee MJ_2013_International J of Infectious Diseases	***	**	*	Selected group of people with TB-DM (presumptive MDR), adjustment for two confounders only, incomplete follow up likely to introduce bias
Nandakumar KV_2013_Plos One	***	***	**	Of 667 TB-DM, exposure status was unknown for 427 (64%)
Park SW_2012_Eur J Clin Microbiol Infect Dis	***	-	*	Excluded extra pulmonary, pulmonary TB with HIV and age <15 years. Adjusted analysis not done, incomplete follow up likely to introduce bias
Tabarsi P_2014_Journal of Diabetes and Metabolic Disorder	****	-	**	adjusted analysis not done
Viswanathan V_2014_Journal of Diabetes and its complications	***	-	**	New smear positive TB cases, adjusted analysis not done
Yoon YS_2017_Thorax	****	-	*	Adjusted analysis done but for not of the comparison of our interest, incomplete follow up likely to introduce bias
Mahishale_2017_Iran J MS	****	***	**	No risk of bias

## Discussion

### Summary of findings

The systematic review identified two studies which provided quality information on glycemic control during TB treatment among people with TB-DM and its effect on TB treatment outcomes. One study provided evidence on reduction in unfavourable end treatment outcomes including recurrence after initial treatment success. [46] The second study provided information on faster TB culture conversion among those with glycemic control. [42] Other studies, though provided some information, were not free of the risk of bias. We did not find a study where less stringent glycemic control’s effect was exclusively assessed on TB treatment outcomes after adjusting for confounders. The two studies on effect of insulin (with or without OHA) on TB treatment outcomes were not free of the risk of bias.

### Qualitative assessment of the studies

Except for Mahishale et al and Magee MJ et al, no study did adjusted analysis to measure the desired associations. Mahishale et al had the largest sample of people with TB-DM (n = 630). Participants were also followed up for two years after treatment initiation to measure recurrence post treatment success. [46] They could have used aRR instead of aOR as the former was most appropriate for the design.[49] One additional HbA1c measurement during treatment, besides baseline) could have helped estimate glycemic control during ATT better. [46] Magee et al used time to event analysis and summarized the association between glycemic control and time to culture conversion using aHR, however the adjustment was done only for drug-resistant status and previous ATT. Despite availability of data, there was no mention of effect (adjusted analysis) of glycemic control, DM care and type of DM treatment on end TB outcomes. The criterion for recording glycemic control was not specified. This study does provide estimates of TB outcome by degree of diabetic care as well as by type of anti-diabetic regimen used unlike Mahishale et al. Unadjusted RR derived from Magee et al revealed worse treatment outcomes among those on insulin. However, we cannot infer much from this as the analysis was unadjusted. [42]

Chiang CY et al’s primary research question did not match ours. But they had analyzed influence of glycemic control on TB treatment outcomes. The HbA1c cut offs used by them <7%, 7–9% and ≥9% did not meet our review’s predefined cut offs, <7%, 7–8% and ≥8%. Of 705 people with TB-DM, 30% had no information on the glycemic control. Even though, HbA1c level was found to be associated (statistically significant) with unfavourable outcomes in unadjusted analysis, they did not include it in the adjusted analysis and the results were summarized as aOR instead of aRR. Non-DM was used as the reference group similar to the multi-centre study by Yoon S et al (n = 157). [40,41]

Nandakumar KV et al (n = 667) reported that control status was ‘known’ for 240 (36%) only. They used very strict operational definitions for ‘known’ diabetic status and control among known. They used RR/aRR which was appropriate for the study design. Multiple blood glucose values were available and the values were summarized into a single variable ‘glycemic control during treatment’. Instead, they could have used the glycemic control data as it is at various points during TB treatment by performing longitudinal data analysis. [44] The same applies to Mi F et al. However, Mi F et al chose to have a cross-sectional comparison: two months glycemic control with end IP outcomes and 6 months glycemic control with end treatment outcomes. [43]

In the study by Park SW (n = 124), unadjusted comparison of culture conversion at 2 months among those with glycemic control (HbA1c <7%) and with poor glycemic control (HbA1c ≥7%) at baseline was made with non-DM as reference. The information was also not reliable as many did not have info on culture conversion. [45] Tabarsi P et al, instead of categorizing into 4 groups, could have used the individual HbA1c value as it is at two time points and looked at its association with end treatment outcome. [47]

The HbA1c test should be performed using a method that is certified by the NGSP (www.ngsp.org) and standardized or traceable to the Diabetes Control and Complications Trial reference assay. [25] With reference to the use of HbA1c in five studies (S1 Annex), there was no mention of NGSP certification or standardized to the DCCT assay.

### Ongoing trials

We have summarized the ongoing trials (n = 5) whose results are awaited in S2 Annex. In all these trials, the primary objective does not meet our review’s objectives. Four trials are looking at effect of Vitamin D supplementation on TB treatment outcomes among people with TB-DM. We hope to get the glycemic control related data from the authors as and when the results are available or published. Another trial is looking at the effect of intensive monitoring of diabetes on diabetes control up to six months during TB treatment when compared to standard diabetes monitoring. Here we hope to get the TB treatment outcome related data from the authors once available.

### Implications for TB-DM management and future research

Among people with TB-DM, this review found a dearth of studies with minimal or no risk of bias for the effect of glycemic control and the effect of insulin (with or without OHA), when compared to OHA only, on TB treatment outcomes. There is a need for RCTs on effect of glucose lowering treatment options on TB treatment outcomes. As of now, the countries may continue to follow the existing guidelines of DM management among people with TB-DM. This also provides an opportunity for the national TB programmes to systematically record glycemic control status at baseline, intensive phase and continuation phase of ATT. Routine reporting and setting up monitoring mechanisms for the same is the need of the hour.

Cohort studies are observational in nature which makes the analysis complex with multiple options. This also makes comparisons and pooling of results complex. In future, for cohort studies on this research question, we recommend a standard methodology which will ensure comparability among the studies. They are as follows: i) conducting a multicenter study which will ensure sufficient number of people with TB-DM; ii) using standard diagnostic criteria to diagnose DM [25]; iii) using TB-DM with uncontrolled glycemic status as the reference instead of TB people without DM; iv) assessment of glycemic status, preferably using HbA1c (standardized), at baseline, end of intensive phase and during continuation phase; v) use of standard HbA1c (7% and 8%) and FBG (130mg/dl and 178 mg/dl) cut offs for glycemic control, thus providing three arms of glycemia (stringent, less stringent, poor control); vi) if three or more FBG values are available during treatment, considering conversion of mean FBG during treatment to an estimated HbA1c during treatment; [25,33] vii) if time of follow up is consistent, then use of RR, and if time of follow up is not consistent and the dates are available, then use of HR as the measure of effect; ix) adjusted analysis for confounders (age, sex, TB site, TB microbiological status, new or old TB, baseline BMI, baseline anemia, HIV status, steroid use, tobacco and alcohol use,); x) ideally, a longitudinal data analysis making use of HbA1c or FBG value at each time point is recommended. This would also adjust for clustering for repeat measurements and not reduce the sample size, which usually happens when people with TB-DM are classified into groups based on baseline and during ATT HbA1c/FBG values. This way we can not only analyze the effect of glycemic control on TB treatment outcomes but also the effect of improving or worsening glycemic control with time.

## Conclusion

This systematic review identified two studies that were free of the risk of bias and suggested glycemic control may have a favourable effect on TB treatment outcomes. However, in many studies, the variables to answer our research question were available, but the analysis was not meeting our review’s objectives. The author group of this systematic review is in touch with the authors of the included studies and is working on a research consortium for either a re-analysis or a pooled analysis of data.

In the future, focused cohort studies are required on this topic, using a standard design and analysis plan. There is a need for RCTs looking at effect of glucose lowering treatment options on TB treatment outcomes.

## Supporting information

S1 Annex Characteristics of studies included in the review. (DOCX)

S2 Annex Characteristics of ongoing studies. (DOCX)

S1 Appendix Search strategy for electronic database search and search results. (7Z)

S2 Appendix Data extraction form containing information of studies included in the review. (7Z)

S3 Appendix PRISMA checklist. (DOC)
