# Supplementary material for: Effect of glycemic control and type of diabetes treatment on unsuccessful TB treatment outcomes among people with TB-Diabetes: A systematic review
Source: PLoS One. 2017 Oct 23;12(10):e0186697. doi: 10.1371/journal.pone.0186697 (PMC5653348; doi:10.1371/journal.pone.0186697)
Supplement: S1 Annex — (DOCX) [file pone.0186697.s001.docx]

**S Annex 1.**Characteristics of studies included in the review

**Study ID: Chiang CY_2015_Plos One**

| Settings | Conducted in a programmatic setting in three teaching hospitals in North South and East Taiwan | |
| --- | --- | --- |
| Methods | Study design: Retrospective cohort  Study duration: 2005-1010  Diabetes diagnosis based on history of treatment with insulin / OHA OR ICD-9 code for DM was given at admission OR ICD-9 code was DM was given twice or more during OPD visit OR h/o DM.  HbA1c was also used for diagnosis (no mention of NGSP certification and standardized to the DCCT assay) | |
| Participants | 705 culture positive pulmonary TB DM patients of all sexes, age not reported irrespective of the HIV status diagnosed with DM within 3 months of initiation of ATT  Included TB -non DM arm with 768 patients | |
| Intervention/ Exposure | Criteria for classification of glycemic control at baseline:  HbA1c<7 – glycemic control arm  HbA1c 7-9-glycemic control – less stringent arm  HbA1c >9 - Poor glycemic control arm | |
| Outcomes | End treatment outcomes- death, treatment failure, loss to follow up | |
| Major conclusions | Poor glycemic control is associated with poor TB treatment outcome and improved glycemic control may reduce the influence of diabetes on TB. | |
| Comments | This paper does not provide direct evidence of effect of glycemic control on TB treatment outcomes among TB-DM patients. First, the comparison throughout the paper was with people without DM. Second, HbA1c values though available were used in the unadjusted analysis, but not in the adjusted analysis.  Adjusted effect of DM (reference was no DM) on TB treatment outcomes was determined before and after including DM related comorbidity (reference was no DM related comorbidity) in the multivariable model. No DM comorbidity included those without DM as well. Finding was that DM related comorbidity resulted in unfavourable TB treatment outcomes; and not DM. It was thus interpreted that hyperglycemia worsened TB treatment outcomes. | |
| **Risk of bias table** |  | |
| Representativeness of exposed cohort (truly representative/somewhat representative/selected group of users/no description of derivation of cohort) | | somewhat representative (of 705 TBDM, 195 (27.7%) info on HbA1c not available |
| Selection of unexposed cohort (drawn from same community as exposed/drawn from a different source/no description) | | same community |
| Exposure ascertainment (secure record or measurement / structured interview / written self-report/ no description) | | secure record or measurement |
| Demonstrated that outcome of interest was not present at start of study (yes/no/not clear) | | yes |
| Adjustment of confounders (age / sex / TB site / TB microbiological status / new or old TB / baseline BMI / baseline anemia / HIV status / Baseline tobacco / baseline alcohol) If yes, how many and which one? | | No (but for a different comparison) |
| Comparability of cohort / confounders Adjustment for other confounders ?mention which one? | | No (but for a different comparison) |
| Adjustment for at least four important confounders (age / sex / TB site / TB microbiological status / new or old TB / baseline BMI / baseline anemia / HIV status / Baseline tobacco / baseline alcohol) Yes / no / not clear | | No (but for a different comparison) |
| outcome assessment (independent blind / record review or linkage / self-report / no description | | record review |
| Adequacy of follow up (complete - all subjects accounted for / loss to follow up unlikely to introduce bias / loss to follow up likely to introduce bias / no statement | | Complete |

**Study ID: Mi F_2013_TMIH**

| Settings | Conducted in a programmatic setting in two TB clinics in Guangzhou Chest Hospital, South China | |
| --- | --- | --- |
| Methods | Study design: Retrospective cohort study  Study duration: September 2011 to June 2012  Diagnosis of DM was done if patient had two FBG measures ≥ 126 mg/dl, one at TB clinic and one at DM clinic | |
| Participants | 189 pulmonary/ extra pulmonary TBDM patients of all age groups, both sexes, HIV status not reported  Included TB non DM arm with 1400 patients | |
| Intervention/ Exposure | Criteria for classification of glycemic control (baseline, 2 months and 6 months)  Glycemic control – good: FBG<7.0 mmol/l  Glycemic control –poor: FBG 7.0- 10 mmol/l  Glycemic control- bad: FBG >10.0 mmol/l | |
| Outcomes | TB treatment outcomes – smear non conversion at 2 months, with favourable outcomes defined as cure and treatment completed and unfavourable outcomes defined as loss-to-follow-up, dead, failure and transfer-out | |
| Major conclusions | **End IP unfavourable outcome- sputum smear positive for acid-fast bacilli at 2 months:**  Comp1:good control vs poor; RR=2.33 (0.8,6.75)  Comp2:good vs bad; RR=1.55 (0.46,5.17)  Comp 3: good v/s poor plus bad; RR=1.97 (0.71,5.47)  (good control is reference)  **End treatment unfavourable outcome- failure**  Comp1:good control vs poor; RR=0.88(0.24,3.20)  Comp2:good vs bad; RR=0.97(0.26,4.69)  Comp 3: good vs poor plus bad; RR=0.91 (0.28, 2.94)  (good control is reference)  Diabetes mellitus in new smear-positive people with PTB was associated with failure to sputum smear convert at 2 months and adverse treatment outcomes of loss-to-follow-up and failure. | |
| Comments | They had glycemic control value at baseline, 2 mon and 6 mon. FBG cut offs (7, 7-10,>10mmol/l) used to define levels of glycemic control did not match with what we have specified in the protocol (7.2mmol/l, 7.2-8.99,>=9)  Though started with 185 people with TB-DM, data on end IP and end treatment outcomes were available for 86 and 77 respectively. And the percentages reported are not based on original cohort but on data availability on outcomes.  Unadjusted crude RRs was based on 2 month FBG and end IP outcome; and 6 month FBG and treatment failed at 6 months (which is cross sectional data). The analysis was cross-section despite a cohort design. Also, the summary measure (RR) used was appropriate for the design; but not for the cross-sectional analysis. | |
| **Risk of bias table** |  | |
| Representativeness of exposed cohort (truly representative/somewhat representative/selected group of users/no description of derivation of cohort) | | no description of derivation of TB DM glycemic control/uncontrolled cohort |
| Selection of unexposed cohort (drawn from same community as exposed/drawn from a different source/no description) | | same community |
| Exposure ascertainment (secure record or measurement / structured interview / written self-report/ no description) | | secure record or measurement |
| Demonstrated that outcome of interest was not present at start of study (yes/no/not clear) | | Yes |
| Adjustment of confounders (age / sex / TB site / TB microbiological status / new or old TB / baseline BMI / baseline anemia / HIV status / Baseline tobacco / baseline alcohol) If yes, how many and which one? | | No |
| Comparability of cohort / confounders Adjustment for other confounders ?mention which one? | | No |
| Adjustment for at least four important confounders (age / sex / TB site / TB microbiological status / new or old TB / baseline BMI / baseline anemia / HIV status / Baseline tobacco / baseline alcohol) Yes / no / not clear | | No |
| outcome assessment (independent blind / record review or linkage / self-report / no description | | record review |
| Adequacy of follow up (complete - all subjects accounted for / loss to follow up unlikely to introduce bias / loss to follow up likely to introduce bias / no statement | | incomplete follow up likely to introduce bias |

**Study ID: Magee MJ_2013_International J of Infectious Diseases**

| Settings | Conducted in a programmatic setting (as a pilot) at primary/secondary level in Lima, Peru | |
| --- | --- | --- |
| Methods | Study design: Retrospective/ prospective cohort- time of data collection unclear  Study duration: Jan2005-May2008  On DM treatment OR all those who reported DM were confirmed by fasting plasma glucose ≥7.0 mmol/l (126 mg/dl) OR 2-h plasma glucose/random blood glucose ≥ 11.1 mmol/l (200 mg/dl) OR ≥ glycosylated hemoglobin (HbA1c) 7%. (no mention of NGSP certification and standardized to the DCCT assay) | |
| Participants | Selected group of TB patients screened for high risk to become MDR TB (people with presumptive MDR-TB)  Included Tb non DM arm of 1485 patients and 186 TB DM patients (pulmonary and EP), new and retreatment aged >=15 years of either sex, regardless of HIV status with DM | |
| Intervention/ Exposure | Based on documentation of control in records/ FBG below median/ FBG<136. Exact criteria not specified  Details on glucose lowering treatment available  OHA only- 56  Insulin only- 16  Both- 26 | |
| Outcomes | End treatment- Failed, default or died  Time to smear conversion- aHR= 2.2(1.1-4.1) where uncontrolled group is reference. This was faster in those with no history of TB previous TB treatment. Frequent DM care also had faster culture conversion but not significant. | |
| Major conclusions | Diabetes was common in a cohort of TB patients at high risk for drug-resistant TB. Despite prevalent multidrug-resistant TB among TB–DM patients, the majority had a favorable TB treatment outcome. | |
| Comments | Analysis was satisfactory (use of aHR). They had baseline and during TB treatment information of the following: glycemic control through HbA1c level, level of diabetes care (scored between 0 and 4) and diabetes medical treatment details. Though TB end-treatment outcomes have been described among TB-DM patients, there was no mention of effect of glycemic control and DM care on TB end-treatment outcomes. Though DM control was objectively measured, it was mentioned that DM control status was based on records (details not clear). | |
| **Risk of bias table** |  | |
| Representativeness of exposed cohort (truly representative/somewhat representative/selected group of users/no description of derivation of cohort) | | selected group of TB-DM (presumptive MDR) |
| Selection of unexposed cohort (drawn from same community as exposed/drawn from a different source/no description) | | same |
| Exposure ascertainment (secure record or measurement / structured interview / written self-report/ no description) | | secure record |
| Demonstrated that outcome of interest was not present at start of study (yes/no/not clear) | | yes |
| Adjustment of confounders (age / sex / TB site / TB microbiological status / new or old TB / baseline BMI / baseline anemia / HIV status / Baseline tobacco / baseline alcohol) If yes, how many and which one? | | Yes, two, prev treatment, drug resistance |
| Comparability of cohort / confounders Adjustment for other confounders ?mention which one? | | Yes |
| Adjustment for at least four important confounders (age / sex / TB site / TB microbiological status / new or old TB / baseline BMI / baseline anemia / HIV status / Baseline tobacco / baseline alcohol) Yes / no / not clear | | No |
| outcome assessment (independent blind / record review or linkage / self-report / no description | | record review |
| Adequacy of follow up (complete - all subjects accounted for / loss to follow up unlikely to introduce bias / loss to follow up likely to introduce bias / no statement | | Incomplete follow up likely to introduce bias |

**Study ID: Nandakumar KV_2013_Plos One**

| Settings | Conducted in a programmatic setting, Primary / Secondary level peripheral health institutions in Malappuram district, Kerala, India | |
| --- | --- | --- |
| Methods | Study design: retrospective cohort  Study duration: April 2010-Sep2011  Diabetes diagnosis: h/o of DM/DM treatment OR FBG≥126 OR RBS≥200 at baseline | |
| Participants | 667 TB DM patients, new or retreatment, pulmonary or extrapulmonary, aged >=15 years, belongin to either sex, irrespective of HIV status | |
| Intervention/ Exposure | Criteria for glycemic control: assessed three times, at least one month apart and at least one in CP. Those with all three values available were classified as ‘known’ glycemic control. Those with all three value less than cut off was ' controlled'  Glycemic cut offs: FBS<100, PPBS/RBS<140 | |
| Outcomes | Sputum non conversion at 2 months : RR=1.14(0.74, 1.75)  End treatment unfavourable outcomes- death, treatment failed, loss to follow up, not evaluated: RR=2.0(0.97, 4.13)  MDR | |
| Major conclusions | Could not confirm an adverse association between DM or its control during treatment and the course of response to TB treatment.DM screening in TB cases and recording of DM care needs to be improved to enable more conclusive evidence. | |
| Comments | Large number of people with ‘unknown’ glycemic control.  Very strict operational definition for ‘known’ glycemic status and ‘control’ glycemic status among those with ‘known’ glycemic status.  Analysis was appropriate | |
| **Risk of bias table** |  | |
| Representativeness of exposed cohort (truly representative/somewhat representative/selected group of users/no description of derivation of cohort) | | somewhat representative (of 667 TBDM, 427 (64%), exposure status was unknown based on their definition) |
| Selection of unexposed cohort (drawn from same community as exposed/drawn from a different source/no description) | | same |
| Exposure ascertainment (secure record or measurement / structured interview / written self-report/ no description) | | secure record |
| Demonstrated that outcome of interest was not present at start of study (yes/no/not clear) | | yes |
| Adjustment of confounders (age / sex / TB site / TB microbiological status / new or old TB / baseline BMI / baseline anemia / HIV status / Baseline tobacco / baseline alcohol) If yes, how many and which one? | | no |
| Comparability of cohort / confounders Adjustment for other confounders ?mention which one? | | no |
| Adjustment for at least four important confounders (age / sex / TB site / TB microbiological status / new or old TB / baseline BMI / baseline anemia / HIV status / Baseline tobacco / baseline alcohol) Yes / no / not clear | | no |
| outcome assessment (independent blind / record review or linkage / self-report / no description | | record review |
| Adequacy of follow up (complete - all subjects accounted for / loss to follow up unlikely to introduce bias / loss to follow up likely to introduce bias / no statement | | complete |

**Study ID: Park SW_2012_Eur J ClinMicrobiol Infect Dis**

| Settings | Study in a clinical setting in two tertiary hospitals Chung-Ang University Hospital and Yong San Hospital, South Korea | |
| --- | --- | --- |
| Methods | Study design: Retrospective cohort  Study duration: Jan 2005- Dec 2009  Diabetes diagnosis based on h/o of DM/DM treatment OR FBG≥126 OR RBS≥200 at baseline | |
| Participants | New pulmonary TB patients of whom were 124 TB DM patients and 368 Tb non DM patients aged >=15 years of either sex who were HIV negative | |
| Intervention/ Exposure | Criteria for glycemic control assessment at baseline  Glycemic control: HbA1c<7  Poor glycemic control HbA1c>=7 | |
| Outcomes | End IP outcome- culture non conversion 2months | |
| Major conclusions | Uncontrolled diabetics seem to have more cavities, higher positive smear rates and lack of culture conversion after two months of therapy. Therefore, TB people with uncontrolled diabetes should be carefully managed and treated. | |
| Comments | Comparison has been made with non-DM as reference. Above information is also not reliable as many did not have info on culture conversion. Crude numbers were extracted in those with and without glycemic control. | |
| **Risk of bias table** |  | |
| Representativeness of exposed cohort (truly representative/somewhat representative/selected group of users/no description of derivation of cohort) | | selected group of users |
| Selection of unexposed cohort (drawn from same community as exposed/drawn from a different source/no description) | | same |
| Exposure ascertainment (secure record or measurement / structured interview / written self-report/ no description) | | secure record |
| Demonstrated that outcome of interest was not present at start of study (yes/no/not clear) | | yes |
| Adjustment of confounders (age / sex / TB site / TB microbiological status / new or old TB / baseline BMI / baseline anemia / HIV status / Baseline tobacco / baseline alcohol) If yes, how many and which one? | | No |
| Comparability of cohort / confounders Adjustment for other confounders ?mention which one? | | no |
| Adjustment for at least four important confounders (age / sex / TB site / TB microbiological status / new or old TB / baseline BMI / baseline anemia / HIV status / Baseline tobacco / baseline alcohol) Yes / no / not clear | | no |
| outcome assessment (independent blind / record review or linkage / self-report / no description | | record review |
| Adequacy of follow up (complete - all subjects accounted for / loss to follow up unlikely to introduce bias / loss to follow up likely to introduce bias / no statement | | Incomplete follow up likely to introduce bias |

**Study ID: Tabarsi P_2014_Journal of Diabetes and Metabolic Disorder**

| Settings | Inpatients in a clinical setting in National Research Institution of Tuberculosis and Lung diseases, Tehran, Iran | |
| --- | --- | --- |
| Methods | Study design: Cohort prospective  Study duration: May 2012-May 2013  Diagnosis of DM based on HbA1c at baseline and 3months. Cut off <6.5% (no mention of NGSP certification and standardized to the DCCT assay)  Normal- normal- No DM  Normal- elevated- uncertain DM  Elevated – Normal- uncertain DM  Elevated – Elevated- DM | |
| Participants | New pulmonary TB patients aged >15 years, of either sex, irrespective of HIV status | |
| Intervention/ Exposure | No attempt to define glycemic control groups | |
| Outcomes | End treatment unfavourable outcomes- death, treatment failed, loss to follow up | |
| Major conclusions | There were changes in HbA1c during the first three-months of anti-TB treatment, but these were not associated with differences in TB treatment outcomes. Transient hyperglycemia should be considered in TB patients and needs to be taken into account in planning care and management. | |
| Comments | Though this study did not exactly fit into our research question(s), we could extract the data that we required. ‘Elevated-normal’ and ‘elevated-elevated’ were included as the study participants relevant to our review. | |
| **Risk of bias table** |  | |
| Representativeness of exposed cohort (truly representative/somewhat representative/selected group of users/no description of derivation of cohort) | | somewhat representative |
| Selection of unexposed cohort (drawn from same community as exposed/drawn from a different source/no description) | | same |
| Exposure ascertainment (secure record or measurement / structured interview / written self-report/ no description) | | secure record or measurement |
| Demonstrated that outcome of interest was not present at start of study (yes/no/not clear) | | yes |
| Adjustment of confounders (age / sex / TB site / TB microbiological status / new or old TB / baseline BMI / baseline anemia / HIV status / Baseline tobacco / baseline alcohol) If yes, how many and which one? | | no |
| Comparability of cohort / confounders Adjustment for other confounders ?mention which one? | | no |
| Adjustment for at least four important confounders (age / sex / TB site / TB microbiological status / new or old TB / baseline BMI / baseline anemia / HIV status / Baseline tobacco / baseline alcohol) Yes / no / not clear | | no |
| outcome assessment (independent blind / record review or linkage / self-report / no description | | record review |
| Adequacy of follow up (complete - all subjects accounted for / loss to follow up unlikely to introduce bias / loss to follow up likely to introduce bias / no statement | | complete |

**Study ID: Viswanathan V_2014_Journal of Diabetes and its complications**

| Settings | Outpatients in a programmatic setting in Five TUs from three districts in TN, India | |
| --- | --- | --- |
| Methods | Study design: retrospective cohort  Study duration: Jan 2011- Mar 2011  Diabetes was diagnosed at baseline based on previous h/o DM OR FPG and 2hPG used but exact criteria not clear  Details of Glucose lowering treatment also elicited | |
| Participants | New pulmonary TB patients, 96 TB DM and 148 Tb non DM, aged >=18 years, of either sex whose HIV status is not reported | |
| Intervention/ Exposure | OHA only n=53, insulin onlyn=18, both n=3, none | |
| Outcomes | End treatment unfavourable outcomes individually death, treatment failed, loss to follow up, not evaluated, MDR Tb | |
| Major conclusions | delayed sputum conversion and high failure rates in TB treatment outcome were common in new smear-positive pulmonary TB people with diabetes. | |
| Comments | Though information on DM treatment was available, sufficient numbers of TB-DM and sufficient numbers of outcomes were not present for us to make any meaningful conclusions | |
| **Risk of bias table** |  | |
| Representativeness of exposed cohort (truly representative/somewhat representative/selected group of users/no description of derivation of cohort) | | somewhat representative |
| Selection of unexposed cohort (drawn from same community as exposed/drawn from a different source/no description) | | same |
| Exposure ascertainment (secure record or measurement / structured interview / written self-report/ no description) | | self-report |
| Demonstrated that outcome of interest was not present at start of study (yes/no/not clear) | | yes |
| Adjustment of confounders (age / sex / TB site / TB microbiological status / new or old TB / baseline BMI / baseline anemia / HIV status / Baseline tobacco / baseline alcohol) If yes, how many and which one? | | no |
| Comparability of cohort / confounders Adjustment for other confounders ?mention which one? | | no |
| Adjustment for at least four important confounders (age / sex / TB site / TB microbiological status / new or old TB / baseline BMI / baseline anemia / HIV status / Baseline tobacco / baseline alcohol) Yes / no / not clear | | no |
| outcome assessment (independent blind / record review or linkage / self-report / no description | | record review |
| Adequacy of follow up (complete - all subjects accounted for / loss to follow up unlikely to introduce bias / loss to follow up likely to introduce bias / no statement | | incomplete follow up unlikely to introduce bias |

**Study ID: Yoon YS_2017_Thorax**

| Settings | Done in a clinical setting in 10 referral hospitals South Korea | |
| --- | --- | --- |
| Methods | Study design: Prospective cohort  Study duration: Sep2012- Sep2014  Diabetes diagnosis based on history or HbA1c≥6.5%  (no mention of NGSP certification and standardized to the DCCT assay) | |
| Participants | New pulmonary TB patients157 TB DM and 504 TB non DM patients, aged 18-75 years of either sex, excluding those with HIV positive | |
| Intervention/ Exposure | Criteria for classifying glycemic control at baseline  Glycemic control: HbA1c<7  Glycemic control-less stringent- HbA1c 7-8.99  Poor glycemic control: HbA1c>=9 | |
| Outcomes | End IP unfavourable- culture non conversion at 2 months  End treatment unfavourable: death, failure, default | |
| Major conclusions | Uncontrolled diabetes is an independent risk factor for poor treatment response in PTB | |
| Comments | The HbA1c cut offs were not as per our protocol. Though mentioned three subgroups of glycemic controls based on HbA1c, they eventually used <7 and≥ 7 at baseline for glycemic control (two subgroups). For adjusted analysis of effect of glycemic control on end IP and end treatment outcomes, reference used was people without DM. OR was used as the summary measure instead of RR. However, we were able to extract the crude numbers for treatment outcomes among the subgroups of glycemic control and people with TB-DM. | |
| **Risk of bias table** |  | |
| Representativeness of exposed cohort (truly representative/somewhat representative/selected group of users/no description of derivation of cohort) | | Some what representative |
| Selection of unexposed cohort (drawn from same community as exposed/drawn from a different source/no description) | | same |
| Exposure ascertainment (secure record or measurement / structured interview / written self-report/ no description) | | secure record |
| Demonstrated that outcome of interest was not present at start of study (yes/no/not clear) | | yes |
| Adjustment of confounders (age / sex / TB site / TB microbiological status / new or old TB / baseline BMI / baseline anemia / HIV status / Baseline tobacco / baseline alcohol) If yes, how many and which one? | | yes but for a different comparison (comparison was between controllled / uncontrolled and non-DM |
| Comparability of cohort / confounders Adjustment for other confounders ?mention which one? | | yes but for a different comparison (comparison was between controllled / uncontrolled and non-DM |
| Adjustment for at least four important confounders (age / sex / TB site / TB microbiological status / new or old TB / baseline BMI / baseline anemia / HIV status / Baseline tobacco / baseline alcohol) Yes / no / not clear | | yes but for a different comparison (comparison was between controllled / uncontrolled and non-DM |
| outcome assessment (independent blind / record review or linkage / self-report / no description | | record review |
| Adequacy of follow up (complete - all subjects accounted for / loss to follow up unlikely to introduce bias / loss to follow up likely to introduce bias / no statement | | incomplete follow up likely to introduce bias |

**Study ID: Mahishale_2017_Iran J MS**

| Settings | Programmatic setting in departments of Pulmonary Medicine, Internal Medicine, Diabetes and Endocrine Department and DOTS centre (TB unit) at a tertiary care hospital, Belgaum, Karnataka | |
| --- | --- | --- |
| Methods | Study design: Prospective cohort  Study duration: Jan 2012- Dec 2014  DM was diagnosed if baseline FBS was more than 126 mg/dl or PPBS more than 200 mg/dl | |
| Participants | 675 new pulmonary TB patients belonging to either sexes, age group unspecified, excluding known HIV positive | |
| Intervention/ Exposure | Glycemic control was defined as baseline  Poor glycemic control- HbA1c>=7%  Optimal glycemic control- HbA1c<7%  (no mention of NGSP certification and standardized to the DCCT assay) | |
| Outcomes | End IP- Smear positivity at 2 months  End treatment- death, default, treatment failed, MDR TB, recurrence | |
| Major conclusions | Poor glycemic control is associated with an increased risk of advanced and more severe TB disease in the form of lung cavitations, positive sputum smear, and slower smear conversion. It has a profound negative effect on treatment completion, cure, and relapse rates in people with pulmonary tuberculosis. | |
| Comments | Used OR instead of RR. Glycemic control was based on baseline HbA1c. Additional HbA1c measurements during treatment would have been helpful | |
| **Risk of bias table** |  | |
| Representativeness of exposed cohort (truly representative/somewhat representative/selected group of users/no description of derivation of cohort) | | truly |
| Selection of unexposed cohort (drawn from same community as exposed/drawn from a different source/no description) | | same |
| Exposure ascertainment (secure record or measurement / structured interview / written self-report/ no description) | | secure record/ measurement |
| Demonstrated that outcome of interest was not present at start of study (yes/no/not clear) | | yes |
| Adjustment of confounders (age / sex / TB site / TB microbiological status / new or old TB / baseline BMI / baseline anemia / HIV status / Baseline tobacco / baseline alcohol) If yes, how many and which one? | | No |
| Comparability of cohort / confounders Adjustment for other confounders ?mention which one? | | No |
| Adjustment for at least four important confounders (age / sex / TB site / TB microbiological status / new or old TB / baseline BMI / baseline anemia / HIV status / Baseline tobacco / baseline alcohol) Yes / no / not clear | | No |
| outcome assessment (independent blind / record review or linkage / self-report / no description | | record review |
| Adequacy of follow up (complete - all subjects accounted for / loss to follow up unlikely to introduce bias / loss to follow up likely to introduce bias / no statement | | Loss to follow up unlikely to introduce bias |
