# Supplementary material for: Effect of glycemic control and type of diabetes treatment on unsuccessful TB treatment outcomes among people with TB-Diabetes: A systematic review
Source: PLoS One. 2017 Oct 23;12(10):e0186697. doi: 10.1371/journal.pone.0186697 (PMC5653348; doi:10.1371/journal.pone.0186697)
Supplement: S2 Annex — (DOCX) [file pone.0186697.s002.docx]

**S Annex 2.** Characteristics of ongoing studies

**Study ID: ChiCTR-IPR-15006395**

| Title | The influence and mechanism of vitamin D3 supplementation on the treatment outcomes of tuberculosis patients of different glucose tolerance |
| --- | --- |
| Principal Investigator / contacts | Wang Qiuzhen, Qingdao University, China  [kevin_1971@126.com](mailto:kevin_1971@126.com) |
| Weblink | <http://www.chictr.org.cn/showproj.aspx?proj=10964> |
| Enrolment status | Ongoing, expected completion by Dec 2018 |
| Study design | Randomized parallel controlled trial |
| Country | China |
| Participants | Pulmonary TB patients with DM |
| Eligibility criteria | Inclusion Criteria:  new diagnosed tuberculosis, sputum smear positive, aged >=18, stable address  Exclusion Criteria:  HIV positive, tumor, pregnant or lactase women, injured lately, adjusted calcium concentration >2.65mmol/L |

**we hope to get details on glycemic control status from this study*

**Study ID: ChiCTR-TRC-12002546**

| Title | The Effect and Mechanism of Retinol and Vitamin A Supplementation in people with diabetes and pulmonary tuberculosis |
| --- | --- |
| Principal Investigator / contacts | Wang Qiuzhen, Qingdao University, China  [kevin_1971@126.com](mailto:kevin_1971@126.com) Aiguo Ma, Qingdao University, China  [maiguo@public.qd.sd.cn](mailto:maiguo@public.qd.sd.cn) |
| Weblink | <http://www.chictr.org.cn/showproj.aspx?proj=7005> |
| Enrolment status | Ongoing, expected completion by Dec 2018 |
| Study design | Randomized parallel controlled trial |
| Country | China |
| Participants | Pulmonary TB people with DM |
| Eligibility criteria | Inclusion Criteria:  The people with pulmonary tuberculosis and diabetes, aged 18~75, diagnosed by the golden criteria of pulmonary tuberculosis and diabetes; no vitamin or mineral supplement one month before the screening.  Exclusion Criteria:  No severe complications of diabetes including diabetic eye diseases, renal disease and foot disease, etc; pregnancy or lactation women; cancer; coronary heart disease; recent suffered trauma or underwent surgery |

**we hope to get details on glycemic control status from this study*

**Study ID: ChiCTR-TRC-10001032**

| Title | The Effect of retinol and vitamin D Supplementation on nutritional state and Treatment Outcome of people with Pulmonary Tuberculosis and diabetes mellitus in China |
| --- | --- |
| Principal Investigator / contacts | Wang Qiuzhen, Qingdao University, China  [kevin_1971@126.com](mailto:kevin_1971@126.com) Aiguo Ma, Qingdao University, China  maiguo@public.qd.sd.cn |
| Weblink | <http://www.chictr.org.cn/showproj.aspx?proj=8506> |
| Enrolment status | Completed |
| Study design | Randomized parallel controlled trial |
| Country | China |
| Participants | Pulmonary TB patients with DM |
| Eligibility criteria | Inclusion Criteria:  1.patient who plan to stay in local place for 2 years; 2. three sputum specimens positive for acid-fast bacilli by direct microscopy and culture; 3. clinical and radiologic signs consistent with pulmonary tuberculosis; 4. no history of previous antituberculosis treatment.  Exclusion Criteria:  1. drug resistance at baseline or during the follow up; 2. extrapulmonary tuberculosis; 3. pregnancy; 4. lactation; 5. use of corticosteroids or supplements containing vitamin A or vitamin D during the previous month; 6. moderate to severe injury or surgery during the previous month; 7. chronic renal failure, liver disease or heart failure. |

**we hope to get details on glycemic control status from this study*

**Study ID: NCT02169570**

| Title | Effect of Supplementary Vitamin D in Patients With Diabetes Mellitus and Pulmonary Tuberculosis (EVIDENT Study): a Randomized, Double Blind, Controlled Trial |
| --- | --- |
| Principal Investigator / contacts | Nadia - Shah, MAS  [nadia.shah@live.com](mailto:nadia.shah@live.com)  Saadiyah Rao, MSc  [dr.saadrao@gamil.com](mailto:dr.saadrao@gamil.com) |
| Weblink | <https://clinicaltrials.gov/ct2/show/record/NCT02169570> |
| Enrolment status | Completed, expected study completion by Dec 2017 |
| Study design | Interventional  Allocation: Randomized  Intervention Model: Parallel Assignment  Masking: Double Blind (Participant, Care Provider, Outcomes Assessor)  Primary Purpose: Treatment |
| Country | Pakistan |
| Participants | Pulmonary TB patients with type 2 DM |
| Eligibility criteria | Inclusion Criteria:  Age 30 to 60 years  Patients having both TB and type 2 DM  Patients consenting to participate  No history of previous ATT  Plane to have ATT and DM treatment  Exclusion Criteria:  Age less than 30 years or greater than 60 years  Pregnant women  Patients having either TB or type 2 DM  Patients refuse to participate  Patients having extra-pulmonary TB or Multi-drug resistant MDR TB or relapse cases  Patients having hepatic or renal diseases or HIV infection  Patients having hypo- or hyper-parathyroidism  Patients on corticosteroids or immunosuppressive or thiazides diuretics or any other drugs known to interfere with vitamin D levels |

**we hope to get glycemic control status from this study*

**Study ID: NCT02106039**

| Title | Concurrent Tuberculosis and Diabetes: Clinical Monitoring, and Microbiological and Immunological Effects of Diabetes During Tuberculosis Treatment |
| --- | --- |
| Principal Investigator / contacts | Hazel Dockrell, Prof  [Hazel.Dockrell@lshtm.ac.uk](mailto:Hazel.Dockrell@lshtm.ac.uk)  Reinout van Crevel, MD, PhD  [reinout.vancrevel@radboudumc.nl](mailto:reinout.vancrevel@radboudumc.nl) |
| Weblink | <https://clinicaltrials.gov/ct2/show/record/NCT02106039> |
| Enrolment status | Ongoing, expected completion by July 2017 |
| Study design | Interventional  Allocation: Randomized  Intervention Model: Parallel Assignment  Masking: No masking  Primary Purpose: Treatment |
| Country | Indonesia, Peru, Romania |
| Participants | Pulmonary TB patients with DM |
| Eligibility criteria | Inclusion Criteria:  adult (> 18 years old) diabetes mellitus patients  diagnosed as having active pulmonary TB  willing to join the study  Exclusion Criteria:  under TB treatment more than 72 hours  steroid-induced or gestational diabetes |
| Experimental arm  Control arm | Intensive monitoring: more intensive monitoring strategy of blood glucose and clinical review  Standard monitoring: glucose monitoring following the prevailing practice at each site |
| Outcome | Better diabetes control in diabetes people with tuberculosis under treatment [Time Frame: Up to 6 months during TB treatment ]  Diabetes control is determined by HbA1c level which will be measured at month 3 and 6 of TB treatment. |

**we hope to get TB treatment outcomes from this study*
